# Diagnostic Value of Serum Creatinine and Cystatin-C-Based Indices and Ishii Score in Cancer-Related Sarcopenia

**DOI:** 10.3390/diagnostics13132179

**Published:** 2023-06-26

**Authors:** Liming Ding, Xingyu Wang, Tiantao Mao, Jibin Li

**Affiliations:** 1 School of Public Health, Chongqing Medical University, Chongqing 400016, China; 13657674198@163.com; 2 People’s Hospital of Wuxi, Chongqing 405800, China; 13594469692@163.com (X.W.); 18223575740@163.com (T.M.)

**Keywords:** cancer, sarcopenia, creatinine, cystatin C, Ishii score chart

## Abstract

Background: Sarcopenia is a key factor affecting the prognosis of cancer patients; however, identifying patients at risk remains challenging. The serum creatinine/cystatin C ratio (CCR) and the sarcopenia index (SI) are new biomarkers for sarcopenia screening. The Ishii test score is an equation based on age, grip strength, and calf circumference for sarcopenia screening. However, their performances in advanced cancer patients have not been thoroughly studied. We aimed to evaluate and compare the accuracy of three screening tools in diagnosing cancer-related sarcopenia. Methods: A total of 215 cancer patients with a median age of 60.5 y were enrolled in this cross-sectional study. The Asian Working Group for Sarcopenia 2019 (AWGS2019) criteria were used as a standard. The diagnostic accuracies of the CCR, SI, and Ishii screening test were analyzed in terms of sensitivity, specificity, negative and positive predictive values, the Youden index, and the receiver operating characteristic (ROC) curve. Results: According to the AWGS2019 criteria, the prevalence of sarcopenia and severe sarcopenia was 47.9% and 18.6%, respectively. The CCR, SI (positively), and Ishii scores (negatively) were correlated with muscle mass. Accordingly, sarcopenia was negatively correlated with CCR and SI, while it was significantly positively correlated with the Ishii score. In males, the AUCs of the CCR, SI, and Ishii scores were 0.743 (95%CI 0.65–0.836), 0.758 (95%CI 0.665–0.852), and 0.833 (95%CI 0.751–0.909), respectively. In females, the AUCs of the CCR, SI, and Ishii scores were 0.714 (95%CI 0.61–0.818), 0.737 (95%CI 0.635–0.839), and 0.849 (95%CI 0.775–0.932), respectively. The AUC of the Ishii score was significantly higher than that of the other screening tools (*p* < 0.001). The cut-off value of the optimal Ishii score was 102.3 (sensitivity: 93.2%, specificity: 59.1%) for males and 98.3 (sensitivity: 93.3%, specificity: 64.7%) for females. Conclusions: The CCR and SI based on serum CysC and creatinine had a remarkably similar overall diagnostic accuracy for sarcopenia in advanced cancer. Among the above three sarcopenia screening tools, the Ishii score chart seemed to have better predictive values of sarcopenia in cancer patients.

## 1. Introduction

Sarcopenia is a progressive, systemic, skeletal muscle disease characterized by low muscle mass, decreased muscle strength, and/or low physical performance [1]. In 2016, the World Health Organization (WHO) included sarcopenia in the International Classification of Diseases (ICD-10), where it was officially recognized as a Category 1 disease with the code M62.84 [2]. Sarcopenia can significantly increase the risk of falls, fractures, disability, and death [3]. The elderly, sedentary or bedridden, patients with chronic wasting diseases, vegetarians, and patients with malignant tumors are the populations with high incidences of sarcopenia.

Sarcopenia can be diagnosed as ‘primary’ or ‘secondary’ according to the causes. Primary sarcopenia refers to a decline in muscle mass and strength due to ageing without other specific cause. Sarcopenia is considered as ‘secondary’ when there are causal factors other than ageing, such as tumor and inflammatory systemic disease, chronic wasting disease, or multiple trauma [4]. Sarcopenia associated with malignancy is called cancer-related sarcopenia.

Cancer patients are at an increased risk of sarcopenia due to the hypercatabolic acceleration of muscle protein decomposition, insufficient energy and protein intake, abnormal hormone secretion causing insufficient muscle protein synthesis, and anti-tumor treatments such as surgery, radiotherapy, and chemotherapy [5]. In a systematic review, the prevalence of sarcopenia in digestive cancers was 43.68% [6]. Research has shown that cancer-related sarcopenia affects patients’ normal metabolism, reduces the efficacy of antitumor drugs, increases postoperative complications, reduces patients’ quality of life, and results in a shorter survival [7,8,9]. In addition, sarcopenia per se is a critical component of cancer cachexia [10,11]. Therefore, the early detection of sarcopenia is needed to guide interventions that might improve the clinical outcomes.

The European Working Group on Sarcopenia in Older People 2 (EWGSOP2) and the Asian Working Group for Sarcopenia 2019 (AWGS2019) revised and improved the definition, diagnostic criteria, and process of sarcopenia. The diagnosis of sarcopenia was based on a comprehensive assessment of three indicators of muscle mass, muscle strength, and physical performance [1,12]. According to the AWGS guidelines for diagnosing sarcopenia, muscle mass measurement methods include dual-energy X-ray absorptiometry (DEXA), computed tomography scanning (CT), magnetic resonance imaging (MRI), and bioelectrical impedance analysis (BIA) [12]. However, the clinical applications of these methods are limited due to the high cost of equipment, their poor mobility, the fact that they cause radiation exposure, and the fact that they are time-consuming [1]. In addition, there is no unified international threshold value for the third lumbar skeletal muscle index (L_3_SMI) in sarcopenia, which is even less suitable for the Asian population. Therefore, there has been an increasing interest in finding simple and reliable tools to evaluate sarcopenia in cancer patients.

In recent years, creatinine (Cr)- and cystatin-C (CysC)-based indices for sarcopenia have attracted attention. Cr and CysC are reliable biomarkers of renal function in clinical practice. Cr is the endogenous product of creatine and phosphocreatine produced in the muscle. CysC is a non-ionic protein that is constantly produced by nucleated cells that are not affected by muscle metabolism. Kashani and colleagues found that the creatinine-to-cystatin-C ratio (CCR) was closely correlated to the paraspinal muscles of L4, as obtained by abdominal CT scanning, and it initially serves as a sarcopenia index for muscle mass estimation [13]. Lien et al. proposed a new sarcopenia index (SI), calculated as serum Cr × eGFR_CysC_ [14]. Recently, a new study showed that the SI could serve as a surrogate biomarker for evaluating sarcopenia in patients with advanced NSCLC [15]. The CCR and SI tend to be simpler and more objective, whereas DEXA, CT, MRI, and BIA measurements are usually time-consuming, inconvenient, and more expensive. The Ishii screening test, based on age, grip strength, and calf circumference, was established by Ishii et al. in 2014 to predict the risk of sarcopenia [16]. Total scores ≥ 105 for men and ≥120 for women are considered as a high-risk of sarcopenia. EWGSOP2 recommends using the Ishii screening test as a case-finding tool before carrying out further testing for sarcopenia confirmation [1]. Studies have shown that the Ishii score has a good diagnostic performance in community-dwelling older adults [17]. The Ishii screening test only requires simple body measurements, and there is no need for the tedious determination of muscle mass and function. However, there have been few studies on the diagnostic accuracy of the Ishii screening test in detecting sarcopenia in cancer patients.

Therefore, the efficacy of the CCR, SI, and Ishii score in cancer patients need further exploration. The aim of this study was to evaluate and compare the accuracy of the three tools in sarcopenia screening in cancer patients.

## 2. Materials and Methods

### 2.1. Study Design and Patients

This cross-sectional study included 245 inpatients admitted to the department of oncology, People’s Hospital of Wuxi, Chongqing from July 2021 to September 2022. The inclusion criteria were: (a) a diagnosis of malignant tumors; (b) no cognitive impairment and good verbal communication; and (c) the ability to complete the entire examinations and tests. The exclusion criteria were as follows: (a) severe renal function decline (eGFR < 60 mL/min/1.73 m^2^); (b) contraindications for BIA; and (c) obvious pitting edema. The study was approved by the Research Ethics Committee of People’s Hospital of Wuxi, Chongqing. Written informed consent was provided by all participants or their statutory representatives.

### 2.2. Data Collection and Anthropometric Measures

Demographic and clinical data were obtained from the medical records of inpatients, including age, sex, tumor type, and tumor stage. Trained researchers measured the participant’s height, weight, calf circumference (CC), handgrip strength (HGS), 6 m walk, and appendicular skeletal muscle. The anthropometric measurements are described briefly as follows: Height and body weight were measured using a height and weight scale. Skeletal muscle mass was measured by using a BIA device InBody270 (Biospace, Seoul, Korea). During CC measuring, the subject took a standing position. The researcher used an inelastic soft ruler to measure the circumference of the strongest part. Handgrip strength and an electronic grip meter EH101 (Xiangshan Company, Zhongshan City, China) were applied to measure the maximum grip strength of the dominant hand twice with a rest interval of 15 s, and the maximum reading was taken for further analysis. A stopwatch was used for measuring the 6 m walking speed, and the walking time was measured. The results of the 6 m walking speed (m/s) were calculated as follows: 6 m divided by the walking time (seconds). During the BIA measuring, the subject wore light clothing, stood barefoot with an emptied bladder and emptied stomach, and had no metal objects such as pacemakers. The skeletal muscle mass index (SMI) was calculated as the skeletal muscle mass divided by the height (m) squared. The body mass index (BMI) was calculated as follows: BMI (kg/m^2^) = body weight/height^2^.

### 2.3. Assessment of Sarcopenia

The commonly used diagnostic criteria for sarcopenia are EWGSOP2 and AWGS2019. A comparison of the diagnostic cut-off points for sarcopenia is shown in Table 1. AWGS2019 was selected as the diagnostic criterion for this study, which involved an Asian population.

According to the AWGS2019 criteria, sarcopenia diagnosing involves an assessment of low muscle mass, low muscle strength, and poor physical performance. Low muscle strength or poor physical performance combined with low muscle mass can be diagnosed as sarcopenia. Those who meet the three criteria were classified as having “severe sarcopenia”. Low muscle mass is defined as SMI < 7.0 kg/m^2^ in men and <5.7 kg/m^2^ in women. Low muscle strength is defined as handgrip strength < 28 kg for men and <18 kg for women. Low physical performance is defined as gait speed < 1.0 m/s [12].

The Ishii score was calculated as follows: for men: 0.62 × (age − 64) − 3.09 × (grip strength − 50) − 4.64 × (calf circumference − 42); for women: 0.80 × (age − 64) − 5.09 × (grip strength − 34) − 3.28 × (calf circumference − 42) [16].

### 2.4. Laboratory Measurements

Blood samples were obtained from the patients in fasting states. Serum Cr and CysC levels were measured using an AU80 automatic analyzer. Serum Cr was determined by the sarcrosine oxidase method. Serum CysC was determined by the latex immunoturbidimetric method. Serum albumin was detected by the bromomefin green method with a Hitachi Labospect-008AS automatic biochemistry analyzer. The Cr/CysC ratio (CCR) in our study was calculated as follows: CCR = [serum Cr (mg/dL)/serum CysC (mg/L)] × 100 [18,19]. According to the Chronic Kidney Disease Epidemiology Collaboration equation, the eGFR_CysC_ was calculated as follows: if serum CysC ≤ 0.8 mg/dL, eGFR_CysC_ = 133 × (CysC/0.8)^−0.499^ × 0.996^Age^(×0.932 if female); if serum CysC > 0.8 mg/dL, eGFR_CysC_ = 133 × (CysC/0.8)^−1.3287^ × 0.996^Age^(×0.932 if female) [20]. The sarcopenia index (SI) was calculated as follows: SI = serum Cr × eGFR_CysC_ [14].

### 2.5. Statistical Analysis

SPSS26.0 and GraphPad Prism8.0 were used for the statistical analysis. Continuous variables were tested by the Shapiro–Wilk test for normality. The variables conforming to a normal distribution were described as mean ± standard deviation, and the differences between the two groups were analyzed by the *t*-test. Variables with non-normal distribution were described by the median (minimum, maximum), and the difference between the two groups was analyzed by the rank sum test. Categorical variables were described by the frequency (percentage), and ANOVA was performed for a comparison among multiple groups. Pearson’s correlation coefficient was used to analyze the correlation between the SMI and CCR, SI, Ishii score, and other indicators. The ROC curve was used to analyze the cut-off values of the CCR, SI, and Ishii score for cancer-related sarcopenia screening. Univariate and multivariate logistic regression were used to analyze the influencing factors of sarcopenia. *p* < 0.05 was considered statistically significant, and *p* < 0.017 (0.05/3) was considered statistically significant in the comparison among different groups.

## 3. Results

### 3.1. Characteristics of the Study Population

A total of 215 cancer patients with a median age of 60.5 years were enrolled in this cross-sectional study. According to the AWGS2019 diagnostic criteria, there were 103 sarcopenia cases (47.9%) out of the cancer patients, including 40 cases with severe sarcopenia (18.6%). The characteristics are shown in Table 2. The SMI, CCR, and SI scores in the sarcopenia group were significantly lower than those of the non-sarcopenia group; accordingly, the Ishii score of the sarcopenia group was significantly higher than that of the non-sarcopenia group (*p* < 0.001). Compared with non-sarcopenia cancer patients, the sarcopenia group patients were older, with lower BMI, HGS, and CC values, and the difference was statistically significant (*p* < 0.05).

### 3.2. Association between the CCR, SI, Ishii Score, and Sarcopenia

**Figure 1:**
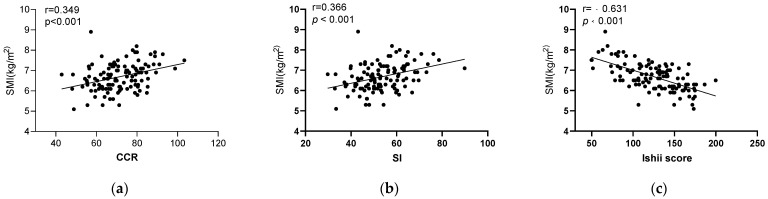

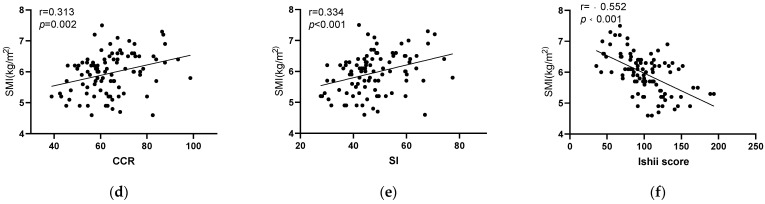
Linear correlation between CCR, SI, Ishii score, and SMI. CCR. (**a**–**c**) Males; (**d**–**f**) females.

The CCR and SI were positively correlated with SMI both in males (r = 0.349 and 0.366, respectively) and females (r = 0.313 and 0.334, respectively), while the Ishii scores were negatively correlated with SMI in males (r = −0.631) and females (r = −0.552), as shown in Figure 1.

**Figure 2:**
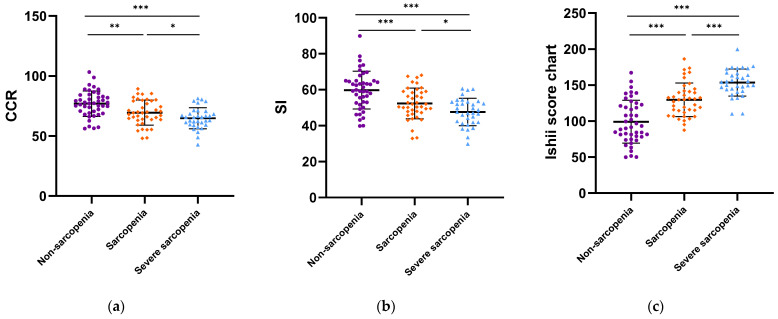

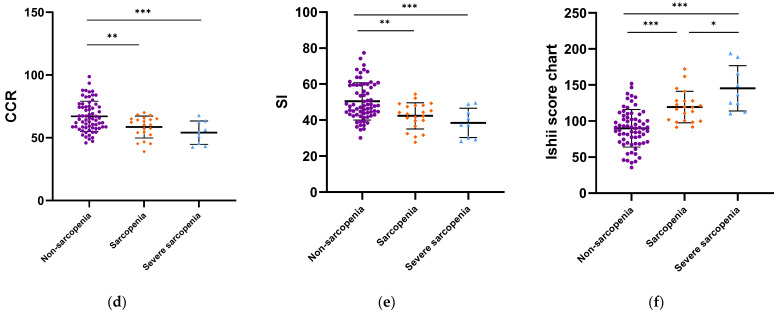
Association of CCR, SI, and Ishii scores with sarcopenia. (**a**–**c**) Males; (**d**–**f**) females. * *p* < 0.05, ** *p* < 0.01, *** *p* < 0.001.

According to the AWGS2019 criteria, the cancer patients were classified into three stage groups: non-sarcopenia, sarcopenia, and severe sarcopenia. As shown in Figure 2, the CCR and SI of the sarcopenia groups were lower than those of the non-sarcopenia groups in both males and females. Meanwhile, the Ishii scores were higher in the sarcopenia groups. In the male groups, the differences in the CCR, SI, and Ishii scores were also statistically significant between the sarcopenia and severe sarcopenia patients (*p* < 0.001).

### 3.3. Optimal Cut-Off Points of the CCR, SI, and Ishii Scores for Sarcopenia Screening

**Figure 3:**
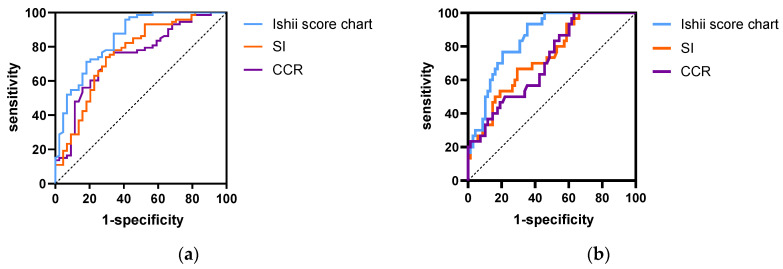
Receiver operator characteristic curves of CCR, SI, and Ishii score for screening sarcopenia according to the AWGS2019 criteria for (**a**) males and (**b**) females.

According to the AWGS2019 criteria, the receiver operator characteristic (ROC) curves of the CCR, SI, and Ishii scores were obtained for males (Figure 3a) and females (Figure 3b). The area under the curve (AUC) was calculated to examine the validity of each screening tool as a predictor of sarcopenia (Table 3). For males, the AUCs of the CCR, SI, and Ishii scores were 0.743 (95%CI, 0.65–0.836), 0.758 (95%CI, 0.665–0.852), and 0.833 (95%CI, 0.751–0.909), respectively. For females, the AUCs of the CCR, SI, and Ishii scores were 0.714 (95%CI, 0.61–0.818), 0.737 (95%CI, 0.635–0.839), and 0.849 (95%CI, 0.775–0.932), respectively. The AUCs of the Ishii scores were significantly higher than those of the CCR and SI (*p* < 0.001).

Subsequently, the optimal cut-off values of each screening tool were analyzed according to the Youden index. As shown in Table 3, the optimal CCR cut-off values were 71.95 for men (sensitivity: 74%, specificity: 70.5%) and 66.5 for women (sensitivity: 86.7%, specificity: 45.6%). The optimal SI cut-off values were 55.34 for men (sensitivity: 74%, specificity: 70.5%) and 44.4 for women (sensitivity: 66.7%, specificity: 70.6%). The optimal Ishii score cut-off values were 102.3 for men (sensitivity: 93.2%, specificity: 59.1%) and 98.3 for women (sensitivity: 93.3%, specificity: 64.7%).

### 3.4. Effects of the CCR, SI, and Ishii Scores as Predictors of Sarcopenia

To analyze the effects of the CCR, SI, and Ishii scores as predictors of sarcopenia, multivariate logistic regression analysis was performed whilst adjusting for gender, age, tumor type, tumor stage, BMI, and Alb.

In model 1, after adjusting for age, sex, diagnosis, tumor stage, BMI, and albumin, the chi-square value of the Hosmer–Lemeshaw test was 5.278, with *p* = 0.727 > 0.05, and the model was well fitted. The multivariate logistic regression analysis showed that the CCR was a predictive factor for sarcopenia, with OR = 0.922 (95%CI, 0.89–0.96; *p* < 0.001), and each unit increase in the CCR was associated with a 7.8% reduction in the risk of sarcopenia. In model 2, after adjusting for age, sex, diagnosis, tumor stage, BMI, and albumin, the chi-square value of the Hosmer–Lemeshaw test was 3.79, with *p* = 0.875 > 0.05, indicating that the model fit well. The multivariate logistic regression analysis showed that the SI was a predictive factor for sarcopenia, with OR = 0.905 (95%CI, 0.86–0.956; *p* < 0.001), and a one-unit increase in the SI was associated with a 9.5% reduction in sarcopenia risk. In model 3, after adjusting for age, sex, diagnosis, tumor stage, BMI, and albumin, the chi-square value of the Hosmer–Lemeshaw test was 5.6, with *p* = 0.692 > 0.05, indicating that the model fit well. The multivariate logistic regression analysis showed that the Ishii score was a risk factor for sarcopenia, with OR = 1.043 (95%CI, 1.02–1.06; *p* < 0.001), and a one-unit increase in the Ishii score was associated with a 4.3% increase in sarcopenia risk. The results are shown in Table 4.

## 4. Discussion

Sarcopenia was initially considered to be a degenerative and systemic skeletal muscle loss due to aging, which can be classified as primary sarcopenia [1]. However, it can also be disease-related, which is recognized as secondary sarcopenia. Particularly in cancer patients, sarcopenia can occur as a result of cancer as well as antitumor therapies. Depending on the cancer type, antitumor therapy, and diagnose methods, the prevalence of cancer-related sarcopenia can be anywhere from 12.5% to 57.7% [21].

Accordingly, 47.9% of the cancer patients in our study were diagnosed as having sarcopenia, which was higher than that of community-dwelling older adults (12.4%) [22]. Given that sarcopenia is associated with many adverse outcomes in cancer patients, early screening could benefit all cancer patients at risk of developing sarcopenia. The COSA (Clinical Oncology Society of Australia) position statement on cancer-related sarcopenia recommended that “All people with cancer should be screened for sarcopenia at diagnosis and repeated as the clinical situation changes, using the validated screening tool.” [23]. In recent years, there has been an increasing interest in assessing new screening tools that can be pragmatically integrated into existing systems.

The AWGS2019 criteria have been widely accepted for diagnosing sarcopenia in clinical occasions. However, skeletal muscle mass determination requires expensive professional equipment or causes radiation exposure. As a result, the application of the AWGS2019 criteria among community dwellers is limited. Therefore, simple and reliable predicting tools are needed. In the present study, we found that the CCR, SI, and Ishii scores were correlated with muscle mass in patients with advanced cancer. After adjusting for confounding factors, the CCR, SI, and Ishii scores independently predicted the presence of sarcopenia. These findings suggested that the three indices can be used as objective tools to assess sarcopenia in clinical oncology.

In recent years, serum biomarkers based on creatinine and cystatin C have been developed as surrogate markers of muscle mass and have been validated in different populations such as intensive care units (ICU) [24], COPD [25], and type 2 diabetes patients [26]. In the present study, we observed the efficacy of the indices among cancer patients. Our results showed that both the CCR and SI were correlated with SMI in male and female patients. This result suggests that the creatinine- and cystatin-C-based indices were valid for muscle mass evaluation in the cancer patients. The CCR and SI showed no significant differences between sensitivity, specificity, PPV, and NPV. However, considering the relatively low coefficient between the CCR and muscle surface area, Lien et al. believed there was room for improvement in the application of the CCR in different clinical conditions [14]. Therefore, they proposed a new index by using timed urine creatinine, which is known as the new sarcopenia index. Because the timed urine creatinine value is dependent solely on muscle mass, the eGFR_CycC_ used in the SI calculation equation is a better way to estimate renal function than creatinine [14]. Nevertheless, in the present study, both the CCR and SI were acceptable in the cancer patients. It is noteworthy that the cystatin C level can be affected by many conditions, such as melanoma [27] and hypothyroid [28]. We did not examine these cases in our study, nor did we analyze the confounding influence of the condition. Further study is needed to address this issue.

The Ishii screening test has been validated in a variety of populations since its establishment [16,29,30,31,32]. This convenient tool makes it possible to rapidly identify individuals at risk of sarcopenia and to improve the opportunity of early diagnosis and intervention. Given these advantages, the Ishii screening test was recommended by EWGSOP2 to be used as a formal instrument for case finding [1]. Considering that the Ishii screening test was developed based on data from Japanese older adults, we performed a validation study in cancer patients. Our results suggested that the optimal Ishii score chart cutoff values were 102.3 for men and 98.3 for women, which were lower than the original cut-off values (105 for men, 120 for women). With these new cut-off values, the Ishii screening test’s sensitivity in predicting sarcopenia increased from 75.5% to 93.3% in females and from 84.9% to 93.2% in males. The increased sensitivity may help clinicians to find cancer patients at risk of sarcopenia. The efficacy of the Ishii score for sarcopenia screening among the cancer patients in this study was similar to that of other studies [29,30,31]. However, the specificity of the Ishii screening test was low compared to the SI, suggesting there was a higher possibility of overestimation of sarcopenia in the present population. The AUC values of the Ishii screening test were also higher (0.833 for males and 0.849 for females) than those of the CCR and SI. The Ishii screening test has displayed similar AUC values in other populations, including community dwellers [17], gastric cancer patients [31], and persons with stroke [30], suggesting that the performance of Ishii screening test is excellent.

Our study has some limitations. First, the study participants were cancer patients from one hospital, so the subjects did not represent all cancer patients in other areas. Further multi-center validation studies are needed in different types of cancer patients. Second, even if BIA is recommended as a method for muscle mass assessment by AWGS 2019, it is less accurate compared to DXA [12,33]. BIA derives an estimate of muscle mass based on whole-body electrical conductivity. Therefore, it is affected by the body hydration status. We excluded patients with edema in the present study. Given that BIA is a valid and convenient tool, more studies are necessary to implement BIA-derived predictions in patients with edema. Third, we did not compare the predictive value of the above tools for prognosis, which should be investigated in future studies.

## 5. Conclusions

The present study found that the prevalence of sarcopenia and severe sarcopenia in tumor patients was 47.9% and 18.6%, respectively. The CCR and SI were positively correlated with the limb skeletal muscle index in cancer patients, while the Ishii score was negatively correlated with the skeletal muscle index of the limbs of cancer patients. The CCR, SI, and Ishii score can be used as simple and feasible indexes for the clinical screening of sarcopenia.

## Figures and Tables

**Table 1:** Diagnostic cut-off points for sarcopenia from EWGSOP2 and AWGS 2019.

Variable	Clinical Practice	EWGSOP2 Cut-Off Points	AWGS2019 Cut-Off Points
Muscle strength	HGS	Male < 27 kg, female < 16 kg	Male < 28 kg, female < 18 kg
	Chair stand test	>15 s for five rises	>12 s for five rises
Muscle quantity	ASM/height^2^	Male < 7.0 kg/m^2^, female < 5.5 kg/m^2^	DXA: male < 7.0 kg/m^2^, female < 5.4 kg/m^2^
			BIA: male < 7.0 kg/m^2^, female < 5.7 kg/m^2^
Physical performance	6 m pace	≤0.8 m/s	≤1.0 m/s
	SPPB	≤8 score	≤9 score

Abbreviations: HGS, handgrip strength; ASM, appendicular skeletal muscle mass; SPPB, short physical performance battery; DXA, dual-energy X-ray; BIA, bioelectrical impedance analysis.

**Table 2:** Demographic and clinical characteristics of the study population.

Variables	All Patients	Non-Sarcopenia	Sarcopenia	*p*
N (%)	215 (100)	112 (52.1)	103 (47.9)	N/A
Age (years)	60.5 (17, 80)	58 (17, 76)	66 (44, 80)	<0.001
Sex				
Male, N (%)	117 (54.4)	44 (37.6)	73 (62.4)	<0.001
Female, N (%)	98 (45.6)	68 (69.4)	30 (30.6)	
Diagnosis				
Digestive system cancer, N (%)	96 (44.7)	42 (43.8)	54 (56.2)	0.028
Others, N (%)	119 (55.3)	70 (58.8)	49 (41.2)	
Tumor stage				
Ⅲ, N (%)	91 (42.3)	54 (59.3)	37 (40.7)	0.068
Ⅳ, N (%)	124 (57.7)	58 (46.8)	66 (53.2)	
BMI(kg/m^2^)	22.03 ± 3.25	23.83 ± 2.84	20.08 ± 2.45	<0.001
HGS(kg)	23.2 ± 6.52	24.6 ± 6.60	21.7 ± 6.10	0.01
CC (cm)	31.5 ± 3.01	33.1 ± 2.65	29.8 ± 2.35	<0.001
Slow gait speed (<1.0 m/s), N (%)	90 (41.8)	24 (26.7)	66 (73.3)	<0.001
SMI (kg/m^2^)	6.35 ± 0.75	6.67 ± 0.71	6.0 ± 0.64	<0.001
Serum Cr (mg/dL)	0.67 ± 0.15	0.67 ± 0.16	0.66 ± 0.15	0.836
Serum CysC (mg/L)	0.99 ± 0.18	0.95 ± 0.17	1.04 ± 0.19	<0.001
eGFR_CysC_ (mL/min.1.73 m^2^)	90.6 ± 19.73	95.0 ± 18.76	85.9 ± 19.75	0.01
CCR	67.8 ± 0.82	70.9 ± 12.46	64.4 ± 10.68	<0.001
SI	50.98 ± 10.83	54.09 ± 11.36	47.59 ± 9.12	<0.001
Ishii score	113.6 ± 34.73	93.5 ± 27.82	135.4 ± 27.63	<0.001

Abbreviations: BMI, body mass index; CC, calf circumference; HGS, hand grip strength; SMI, skeletal muscle index; Cr, creatinine; CysC, cystatin C; eGFR_CysC_, estimated glomerular filtration rate from serum cystatin C; CCR, serum creatinine/cystatin C ratio; SI, sarcopenia index.

**Table 3:** Optimal cut-offs of CCR, SI, and Ishii scores for sarcopenia screening in men and women.

Screening Tools	AUC% (95%CI)	Cut-Off Value (Points)	Sensitivity (%)	Specificity (%)	PPV (%)	NPV (%)	Youden Index
CCR							
Male	0.743 (0.65–0.836)	71.95	74	70.5	80.6	62	0.445
Female	0.714 (0.61–0.818)	66.5	86.7	45.6	41.27	88.57	0.323
SI							
Male	0.758 (0.665–0.852)	55.34	74	70.5	80.6	62	0.445
Female	0.737 (0.635–0.839)	44.4	66.7	70.6	50	82.76	0.373
Ishii score							
Male	0.833 (0.751–0.909)	102.3	93.2	59.1	79.07	83.87	0.523
Female	0.849 (0.775–0.932)	98.3	93.3	64.7	52.83	95.6	0.580

Abbreviations: AUC, the area under curve; CI, confidence interval; NPV, negative predictive value; PPV, positive predictive value; CCR, creatinine/cystatin C ratio; SI, sarcopenia index.

**Table 4:** Relationship between CCR, SI, Ishii scores, and sarcopenia in multivariate-adjusted logistic regression models.

Variables ^1^	Model 1		Model 2		Model 3	
	OR(95%CI)	*p* Value	OR(95%CI)	*p* Value	OR(95%CI)	*p* Value
Sex	4.509 (1.64–12.38)	0.003	5.013 (1.80–13.96)	0.002	1.480 (0.63–3.47)	0.367
Age (years)	1.073 (1.03–1.12)	0.001	1.044 (0.99–1.09)	0.056	1.020 (0.97–1.07)	0.421
Diagnosis	0.639 (0.28–1.46)	0.286	0.677 (0.30–1.54)	0.351	0.728 (0.31–1.69)	0.460
Tumor stage	0.801 (0.36–1.81)	0.594	0.828 (0.37–1.87)	0.649	0.376 (0.15–0.92)	0.032
BMI	0.624 (0.54–0.73)	<0.001	0.617 (0.53–0.72)	<0.001	0.667 (0.57–0.79)	<0.001
Albumin (g/L)	1.005 (0.92–1.10)	0.91	1.004 (0.92–1.10)	0.936	0.999 (0.91–1.09)	0.974
CCR	0.922 (0.89–0.96)	<0.001	-	-	-	-
SI	-	-	0.905 (0.86–0.95)	<0.001	-	-
Ishii	-	-	-	-	1.043(1.02–1.06)	<0.001

^1^ Included variables with *p* < 0.10 in univariate analysis.

## Data Availability

Not applicable.
